# Early response of methanogenic archaea to H_2_ as evaluated by metagenomics and metatranscriptomics

**DOI:** 10.1186/s12934-021-01618-y

**Published:** 2021-07-03

**Authors:** Balázs Kakuk, Roland Wirth, Gergely Maróti, Márk Szuhaj, Gábor Rakhely, Krisztián Laczi, Kornél L. Kovács, Zoltán Bagi

**Affiliations:** 1 Institute of Medical Biology, University of Szeged, Szeged, Hungary; 2 Department of Biotechnology, University of Szeged, Szeged, Hungary; 3 Institute of Plant Biology, Biological Research Center, Szeged, Hungary; 4 Institute of Biophysics, Biological Research Center, Szeged, Hungary; 5 Department of Oral Biology and Experimental Dental Research, University of Szeged, Szeged, Hungary

**Keywords:** Hydrogen, Biomethane, Anaerobic digestion, Methanogenesis, Hydrogenotrophic methanogens, Metagenome, Metatranscriptome, Renewable energy, Power-to-Gas

## Abstract

**Background:**

The molecular machinery of the complex microbiological cell factory of biomethane production is not fully understood. One of the process control elements is the regulatory role of hydrogen (H_2_). Reduction of carbon dioxide (CO_2_) by H_2_ is rate limiting factor in methanogenesis, but the community intends to keep H_2_ concentration low in order to maintain the redox balance of the overall system. H_2_ metabolism in methanogens becomes increasingly important in the Power-to-Gas renewable energy conversion and storage technologies.

**Results:**

The early response of the mixed mesophilic microbial community to H_2_ gas injection was investigated with the goal of uncovering the first responses of the microbial community in the CH_4_ formation and CO_2_ mitigation Power-to-Gas process. The overall microbial composition changes, following a 10 min excessive bubbling of H_2_ through the reactor, was investigated via metagenome and metatranscriptome sequencing. The overall composition and taxonomic abundance of the biogas producing anaerobic community did not change appreciably 2 hours after the H_2_ treatment, indicating that this time period was too short to display differences in the proliferation of the members of the microbial community. There was, however, a substantial increase in the expression of genes related to hydrogenotrophic methanogenesis of certain groups of Archaea. As an early response to H_2_ exposure the activity of the hydrogenotrophic methanogenesis in the genus *Methanoculleus* was upregulated but the hydrogenotrophic pathway in genus *Methanosarcina* was downregulated. The RT-qPCR data corroborated the metatranscriptomic

**Results:**

H_2_ injection also altered the metabolism of a number of microbes belonging in the kingdom Bacteria. Many Bacteria possess the enzyme sets for the Wood-Ljungdahl pathway. These and the homoacetogens are partners for syntrophic community interactions between the distinct kingdoms of Archaea and Bacteria.

**Conclusions:**

External H_2_ regulates the functional activity of certain Bacteria and Archaea. The syntrophic cross-kingdom interactions in H_2_ metabolism are important for the efficient operation of the Power-to-Gas process. Therefore, mixed communities are recommended for the large scale Power-to-Gas process rather than single hydrogenotrophic methanogen strains. Fast and reproducible response from the microbial community can be exploited in turn-off and turn-on of the Power-to-Gas microbial cell factories.

**Supplementary Information:**

The online version contains supplementary material available at 10.1186/s12934-021-01618-y.

## Background

Anaerobic digestion (AD) of organic wastes and by-products by specialized microbial communities and the concomitant biogas production is an environmentally attractive bioenergy production technology. In the context of climate change, the generation of biogas as a renewable energy form has become popular and intensively examined over the last few decades [1].

Biogas provides environmental benefits with regard to waste treatment, pollution reduction, production of CO_2_-neutral renewable energy and the improvement of economy of agricultural practices through the recycling of plant nutrients and replacing artificial fertilizers [2].

Biogas can be burnt to produce heat or combusted in gas engines for electricity generation and, after purification, it can be used in any application for which fossil fuel natural gas is utilized today [3]. AD is applicable to a wide range of waste streams derived from the agro-food industry, which is a source of vast amounts of readily degradable organic material composed mainly of complex organic molecules, as well as in liquid or solid communal waste treatments.

While the main microorganisms and mechanisms involved in the methane producing anaerobic microbial cell factories are fairly well-known, the regulation and management of the overall process is far from being fully understood [4, 5]. Despite the industrial-economic importance of the underlying microbiological events, little is known about the roles, networking interactions of the microorganisms and the regulatory mechanisms of the methane production. Therefore, the microbiological events representing the bottlenecks of the process are difficult to manage. AD demands the concerted action of a complex community of microbes, each member performing their special role in the overall degradation process [6, 7]. In the absence of terminal electron acceptors such as nitrate, oxygen or sulfate, the methanogenic conversion of organic matter is an essential feature of many ecosystems [8].

H_2_ metabolism is one of the most important rate-limiting processes in methanogenesis. H_2_-coupled electron transfer has been verified as an important extracellular pathway of sharing reducing equivalents within the anaerobic environment, especially in microbial electrosynthesis systems [9].

H_2_ conversion is performed at molecular level by the class of enzymes called hydrogenases. Several hydrogenases have been identified in methanogenic archaea their brief overview is appropriate here. The series of reactions involved in methane (CH_4_) formation from H_2_ and carbon-dioxide (CO_2_) are initiated by the formylmethanofuran dehydrogenase. This enzyme catalyzes the formation of N-carboxymethanofuran from methanofuran and CO_2_ [10]. The electrons from H_2_ are first taken up by coenzyme F_420_, which is embedded in the enzyme F_420_-dependent hydrogenase. The reduced coenzyme F_420_ is the central electron carrier in methanogenic archaea. Other hydrogenases from methanogens cannot reduce F_420_ [11]. Methanogenesis from formate involves oxidation of the substrate to produce CO_2_ and a reduced electron carrier. The reaction is catalyzed by a formate dehydrogenase [12]. A novel hydrogenase (Ech) was discovered in acetate-grown cells of *Methanosarcina barkeri,* which shows sequence homologies to hydrogenases 3 and 4 of *Escherichia coli* and to the CO-induced hydrogenase from *Rhodospirillum rubrum*. The purified enzyme from *Ms. barkeri* catalyzed the H_2_-dependent reduction of a 2[4Fe-4S] ferredoxin and is also able to perform the reverse reaction, namely, H_2_ formation from reduced ferredoxin [13]. Some hydrogenases are components of the H^+^-translocating system in methanogens [14]. The effect of H_2_ on the expression of genes coding for hydrogenases and other methanogenesis genes has not been systematically examined yet. It is astonishing to note the complexity of the molecular machinery, which handles the simplest molecule, H_2_. The exploration of the molecular networks, which affect the expression of these genes could improve our extended knowledge concerning molecular redox mechanisms in microbial cell factories.

AD is one of the most promising among the various biomass conversion processes. The regulatory roles of the H_2_ levels have been recognized as a significant element in the concerted action of the complex microbial community [14, 15]. We demonstrated earlier that by the introduction of H_2_-producing bacteria into a natural biogas-generating consortium appreciably increased the efficacy of biogas production both in batch fermentations and in scaled-up continuous AD [14]. One of the rate-limiting factors of AD is the actual level of H_2_ in the system [16]. The presence of excessive amounts of H_2_ inhibits the activity of the acetogenic bacteria that generate H_2_ in the system, whereas limiting H_2_ levels have an adverse effect on an important group of methane producing Archaea, the hydrogenotrophic methanogens. In natural ecosystems, a very low partial pressure of H_2_ is maintained, which may be a limiting factor for the methanogenesis [8, 17]. The relationship between the acetogens and methanogens is syntrophic, supported by a process called interspecies hydrogen transfer or interspecies electron flow [18]. We have only incomplete information about the detailed mechanism of interspecies hydrogen transfer [19]. The actual H_2_ concentration has been shown to determine the composition of the methanogenic community [20–22]. The expression of up to 10% of the total proteins in a hydrogenotrophic methanogen were reported to change in response to H_2_ limitation [23], indicating that the H_2_ availability is sensed by the methanogens and this gas has a major effect on their physiology.

Metagenomic analyses offer a new toolbox for the investigation of the complex microbial cell factories. The reconstruction of the genomes (metagenome assembled genomes: MAGs) of the individual members of a complex microenvironment and their subsequent functional and phylogenetic analysis is termed genome-centric metagenomics [24, 25]. Genome-centric metagenomics (referred to as MG hereafter) already yielded valuable insights into the functional organization of biogas reactors and the microbial cell factories operating within [26, 27]. Additionally, its combination with metatranscriptomics (the analysis of the whole microbial community mRNA in a microenvironment), i.e., genome-centric metatranscriptomics (referred to as MTR, hereafter) enables the examination of the gene expression of each individual MAG, has been used for the in-depth analysis of the process control, regulation and interactions among the members of these cell factories.

In previous approaches the consequences long-term and/or steady H_2_ exposure have been investigated [28–31]. This study is dedicated to unveil the early response of the anaerobic mixed microbial consortium, with special emphasis on methanogens to the presence of H_2_ distress. This is a realistic scenario in large scale AD plants due to local concentration gradients as well as in natural environments, e.g. in swamps or rice fields. More importantly, a quick turn-on and turn-off of H_2_ supply can be expected in the Power-to-Gas technologies, where the fluctuating production of renewable electricity, e.g. by photovoltaic or wind power, is coupled with its biological conversion to biomethane [17, 21]. The central challenge to be understood is the regulatory role of H_2_ in CH_4_ formation and the early response by the methanogens and other H_2_-metabolizing microbes, which regulates and balances the fragile bioenergetic processes in AD.

## Results

### Fermentation

A constant value of VOA/TIC is a reliable indicator of a stable mesophilic fermentation process [32]. Each experiment started with a 20 days long start-up period in order to adapt the microbial community to the alpha-cellulose substrate. During this period the average VOA and TIC values stabilized at VOA = 1.1 g L^−1^ and the TIC = 14 g CaCO_3_ L^−1^. Because of the relatively low substrate loading rate, the VOA/TIC ratios were moderate, which allowed balanced operations. The amount of NH_4_^+^ is also an important indicator of AD process stability [33]. Theoretically, levels above 3000 mg NH_4_^+^ L^−1^ may have a negative effect on the methanogenic archaea, which is the most sensitive group of microbes in the AD process [34]. The NH_4_^+^ concentration was below 1000 mg L^−1^ during the whole fermentation process. The biogas productivity of the digesters was also stable: 650 mL_N_ biogas alpha-cellulose g^−1^ day^−1^ were produced with 53% of CH_4_ content. The first samples for DNA and RNA analysis were taken on day 20 from the stabilized reactors. After sampling the digesters were flushed with H_2_ gas from a gas cylinder for 10 min and 2 h later the second sampling was carried out. This protocol was repeated after 2 months of reactor operation.

The reactors displayed stable operation during the course of the experiment. The daily biomethane production varied by < 10%. The H_2_ injection took place on days 15 and 71 (blue dotted arrows in Additional file 1: Figure S1).

The reactors responded with a sudden increase in daily CH_4_ evolution by 20–25% at both time points, which lasted for 1–2 days (Additional file 1: Figure S1). The CH_4_ content of the biogas was 53% throughout the experimental period. Afterwards the reactors returned to their previous biomethane production levels. It is worth noting that the microbial community responded exactly the same manner to the H_2_ spike 2 months apart, which indicates the robustness, reproducibility and quick response time by the microbial community. Assuming H_2_ saturation of the liquid phase by the 10 min long H_2_ bubbling, we estimated that more than 95% of the injected H_2_ was converted to CH_4_ by the community within 16–24 h, although the amount of available dissolved H_2_ decreased rapidly during the second half of the H_2_ consumption phase. This was in line with the observations of Szuhaj et al. [35], who found in fed-batch H_2_ feeding experiments at much lower scale that the injected H_2_ was completely consumed in 16–24 h. The H_2_ injection apparently did not alter markedly the cumulative biomethane production curve, which showed a straight line throughout the experiment.

### Genom-centric metagenome and metatranscriptome analyses

In the early response of the residing microbial consortium to the sudden H_2_ burst at transcriptome level of metagenome-assembled genomes (MAGs) [36] it was anticipated that the microbial composition and the relative abundances of species did not change substantially within 2 h, i.e., sampling before and after H_2_ exposure. An extensive binning procedure became possible as the number of metagenomic samples elevated. Therefore the H_2_ triggered differences in the gene expression levels could be precisely assessed together with associated alterations in cell physiology.

**Fig. 1:**
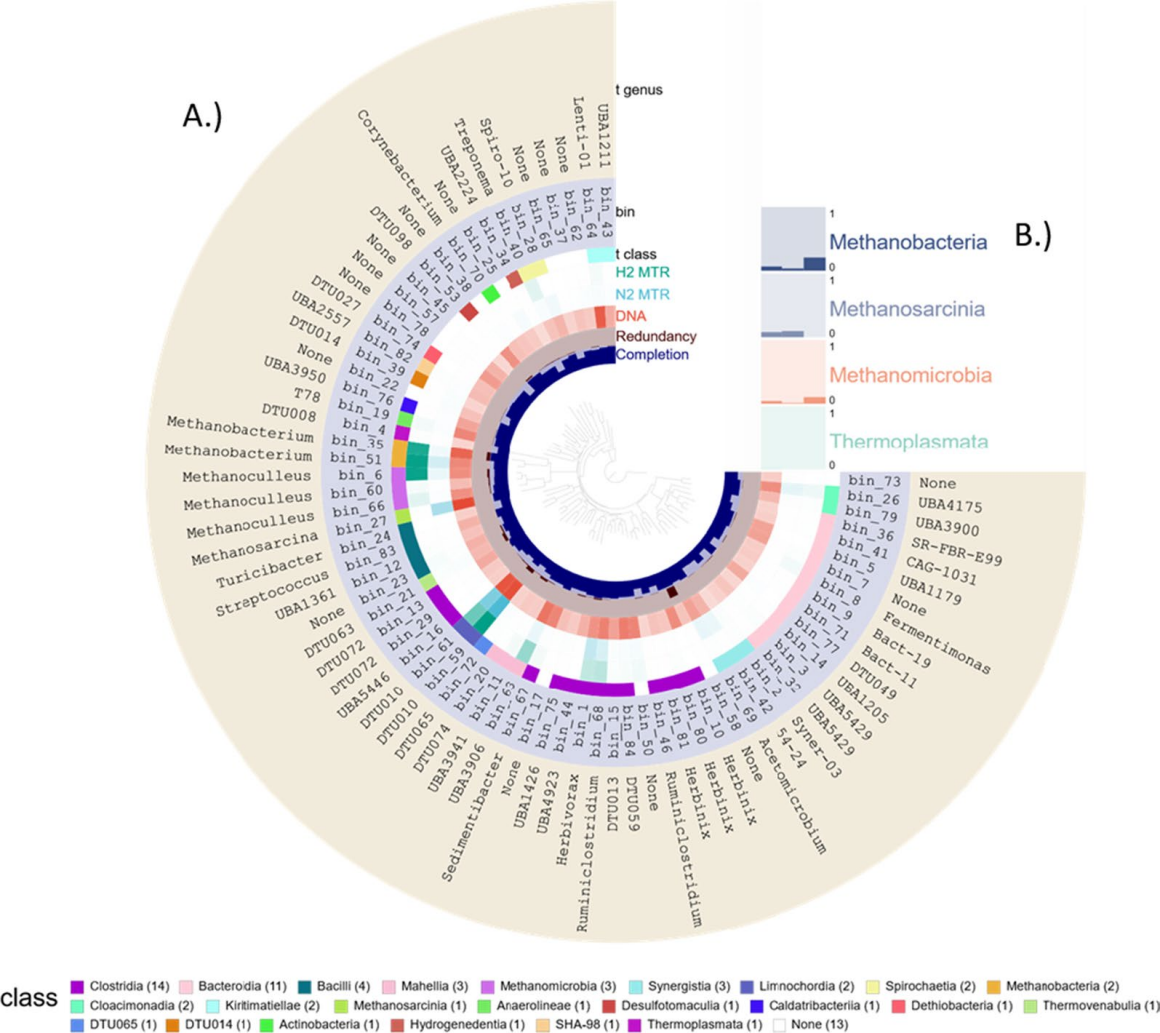
**A** Anvi’o plot of binning results (from innermost to outermost): phylogenetic relationship of bins according to phylophlyan3; completion and redundancy of the bins, according to single-copy marker gene (SCG) content; taxonomic Class and Genus assignment for the bins and relative abundance of bins in samples. The list of Classes at the bottom part indicates the color code and the number of bins in the Classes. **B** depicts the relative abundance of Archaeal Classes (the summary of bins in the Classes)

The extensive binning procedure on the co-assembled contigs and read mapping, employing the three metagenomic binners and the DAS tool, yielded 84 bins. Out of these, 16 were high, 49 were medium and 19 were low quality, according to the MIMAG initiative [36]. 73 bins harbored enough single copy marker genes (SCG) for the phylogenetic tree building (center part in Fig. 1)—the phylogenetic relationship of the remaining 11 bins could not be determined probably because of the low quality of the metagenomes.

The taxonomic assignment of the 84 bins (or MAGs as both of these synonymous expressions will be used in this discussion) resulted in seven Archaea, 61 Bacteria and 16 unclassified bins (details are compiled in Additional file 5: Table S1). Archaea represented about 10% of the microbiome. Within the domain Bacteria, most bins (34) were associated with the phylum *Firmicutes*. The dominance of *Firmicutes* in biogas reactors is in accordance with previous studies [8, 26]. This can be attributed to their diverse capability in polysaccharide and oligosaccharide degradation, which is the first step in the AD of complex organic substrates [37].

The second well-represented phylum was *Bacterioidetes* (12 bins), all of them belonged in the order *Bacteroidales*. Most *Bacterioidetes* produce succinic acid, acetic acid, and in some cases propionic acid, these molecules fuel the acetotrophic methanogenesis. In addition, representatives of the phyla *Synergistota*, *Spirochaetes*, *Verrucomicrobia*, *Cloacimonadota*, *Fibrobacterota*, *Caldatribacteria*, and *Chloroflexota* were identified (Additional file 5: Table S1). The overall microbial landscape is in line with previous studies [8, 26]. A typical microbial community flourished in our biogas digesters, which indicated that the synthetic medium containing only cellulose as a carbon source proved to be a good model system for the metatranscriptomic investigations [38]. This has been corroborated in a comparison of our 84 bins with the MAGs library compiled in Bio-Gas Microbiome database (https://microbial-genomes.org) (Additional file 5: Table S1) [39]. The comparison of the coding sequences, i.e., fasta files, revealed the counterparts of 70 of our 84 MAGs in the Bio-Gas Microbiome database, 56 of the MAGs had more than 95% similarity. The remaining bins contained medium or low quality metagenomes, which could be the main reason for not finding more matches. Two high quality bins (bin_1—*Herbivorax saccincola* and bin_35—*Methanobacterium* sp.) was apparently not represented among the nearly 1600 species identified in Bio-Gas Microbiome.

**Fig. 2:**
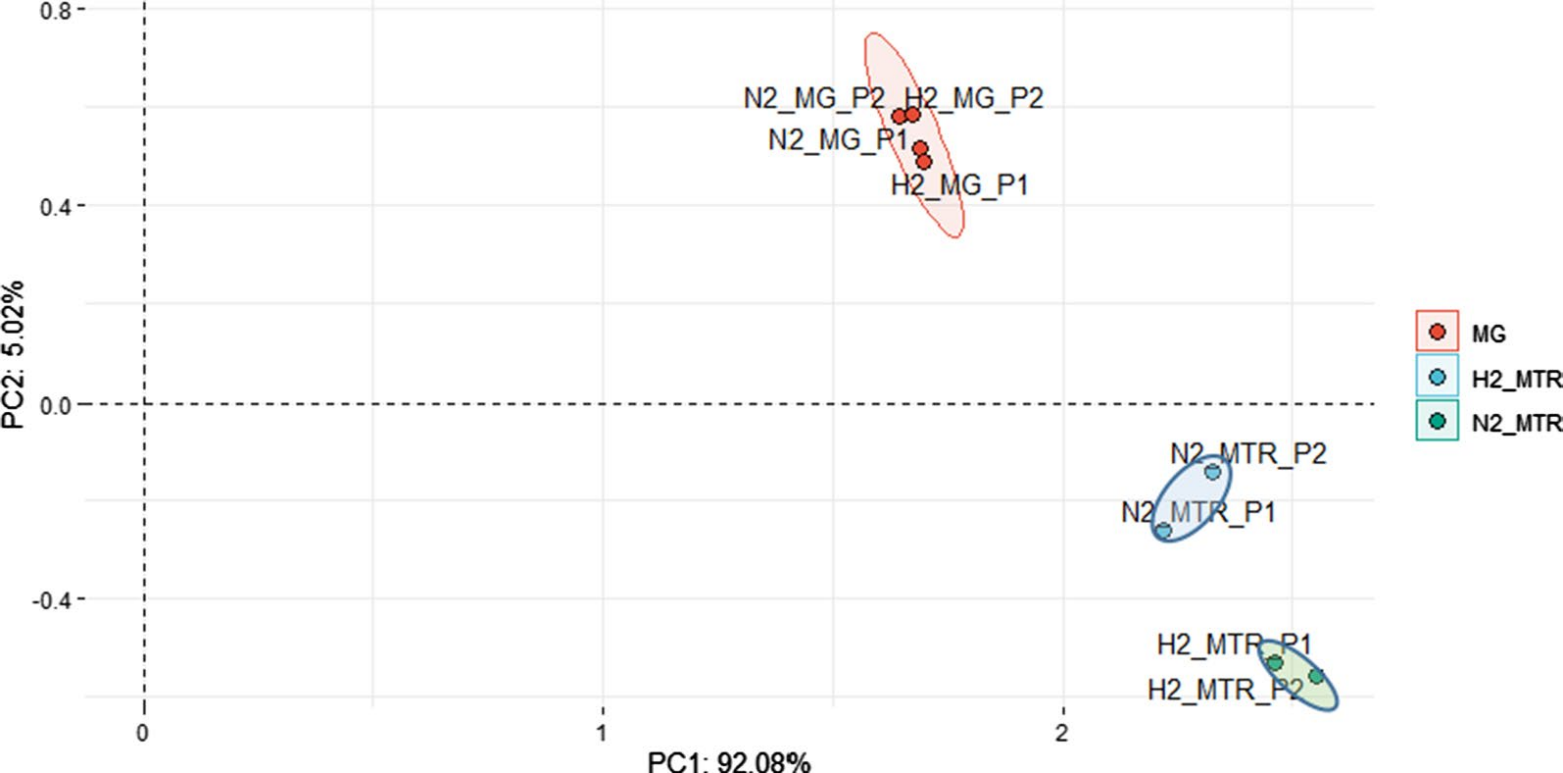
PCA biplot of the rlog-transformed (regularized-logarithm transformation) total gene expressions, i.e., copy number in the MG sample, of each MAG in each sample

A comparison of the DNA-based omics data clearly indicated that the community compositions were very similar in all four samples (Fig. 2), respectively (Additional file 5: Table S1). The overall Archaea gene abundance, i.e., sum of read counts, was 18.49 ± 2.04% in N2-MG or H2-MG samples (each DNA-based). This observation corroborates that (i) all reactors that worked under the same conditions maintained the same microbial community; (ii) as expected, the microbial communities did not change perceptibly within 2 h; and (iii) the observations were highly reproducible after 2 months. In contrast, the mRNA-based metatranscriptome analysis showed striking changes in the transcriptome-based community composition when H_2_ was offered to the reactors’ microbial community. The N2_MTR samples (RNA-based, before H_2_ addition) showed a similar total Archaea abundance to that of the MG samples: 18.99 ± 11.64%, but this was elevated to 36.53 ± 3.74% in the case of H2_MTR samples (RNA-based, after H_2_ addition). This demonstrates a rapid response to the appearance of excess H_2_.

The elevation of the total number of transcribed Archaea genes (H2_MTR samples) was mainly attributed to representatives of the genus *Methanobacterium* (bins 35 and 51), which increased from 4.33 to 17.39% (log_2_FC = 2.84) of all bins’ abundance. *Methanobacteria* are hydrogenotrophic methanogens. The second major contributor to Archaea transcripts was the order *Methanomicrobiales*, from 2.69 to 7.03% (log_2_FC = 2.16). The genera *Methanoculleus* and *Methanosarcina* both belong in this order. The three bins of the genus *Methanoculleus* showed elevated overall abundance, the log_2_FC values of bin_6, bin_60 and bin_66 were 3.64, 2.37 and 2.18, respectively. The increase upon H_2_ exposure was the most apparent in the case of bin_6. *Methanoculleus_bourgensis,* whose proportion increased from 1.65 to 10.66%. Remarkably, the genus *Methanosarcina* effectively ceased to express genes to near zero upon H_2_ dispensation. *Methanosarcina* are known to possess genes coding for all three methanogenic pathways, i.e., hydrogenotrophic, acetotrophic and methylotrophic methanogenesis [8, 16]. Members of the genus *Methanoculleus* are solely hydrogenotrophic methanogens. H_2_ exposure apparently turns on the activity of the hydrogenotrophic methanogenesis in both *Methanoculleus* and *Methanobacterium* but turns off the hydrogenotrophic pathway in *Methanosarcina*.

### Metatranscriptomic pathways analysis

**Fig. 3:**
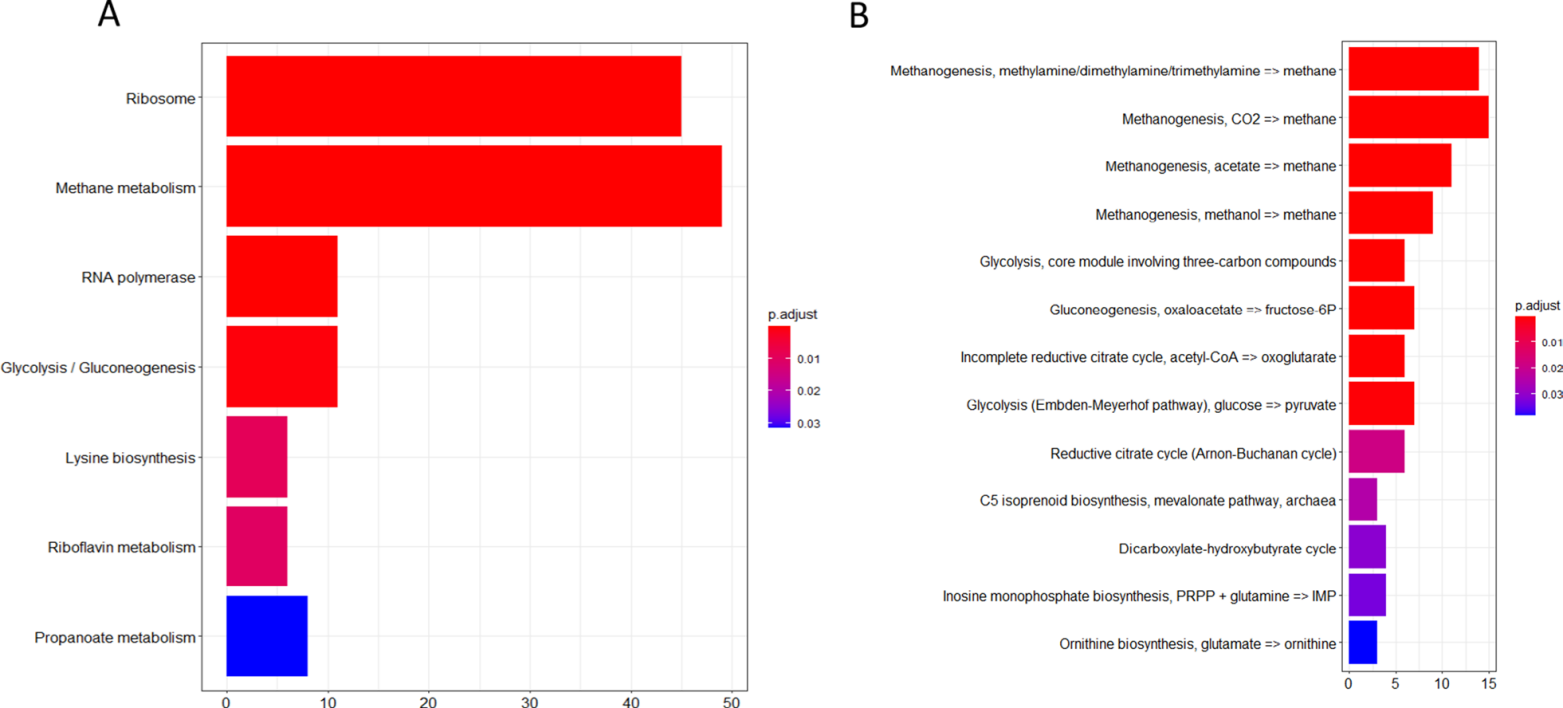
**A** Results of KEGG Module enrichment analysis (left), and **B** KEGG Pathway (right). The pathways, which were significantly different between N2_MTR and H2_MTR samples are presented. X-axis indicates the number of KEGG IDs found as significantly different in the given pathway (listed along the Y axis). P-adjust stands for corrected p-values

A community-level pathway enrichment analysis was performed to examine the overall metatranscriptomic changes that occurred as a result of the H_2_-addition. The contig assembly and ORF prediction/annotation workflow yielded 219,353 KEGG Orthology (KO) annotated ORFs. Out of these 98,791 ORFs were binned in the refined MAGs. The remaining 120,562 ORFs were used for the community-level pathway analysis. The changes in the expression levels of the genes involved in the various methanogenesis related metabolic pathways and modules were examined according to KEGG annotation. The results indicated that the methanogenesis pathway was primarily affected as the result of H_2_ injection (Fig. 3). The upregulation of differentially expressed (DE) genes was the highest in this pathway (48) and in the associated modules. It is noteworthy that some other carbon metabolism associated pathways were also affected, such as Glycolysis/Gluconeogenesis and Propanoate metabolism, which suggest that acetogenic and acetate utilizing microbes were also affected by the specifically altered environment. H_2_ is known to inhibit acetogenic microbes [40], thus their response to the H_2_ addition is not surprising. The RNA polymerase pathway also changed significantly, this was due to triggered transcription machinery as a response to the altered environment.

Despite the binning efforts, many KEGG annotated genes remained unbinned (Additional file 2: Figure S2). Omitting these from the downstream analysis would have distorted the pathway and statistical analyses, therefore we combined them as a group of “unbinned” genes.

### Changes in the expression levels in methanogenesis genes

**Fig. 4:**
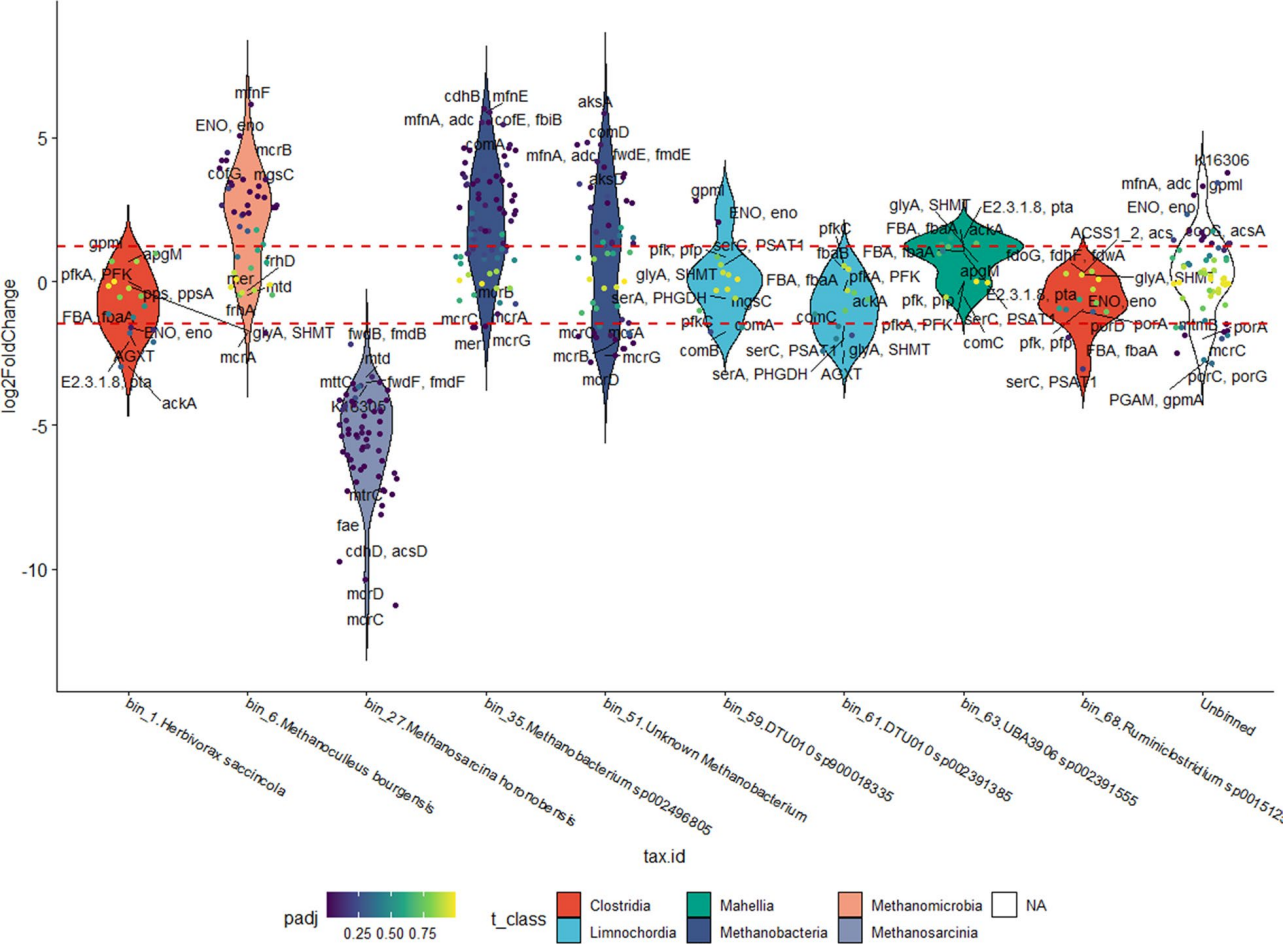
Violin plot of genes (small dots) involved in the methanogenesis KEGG pathway (map00680) in each bin (arranged on the X-axis) and the unbinned gene collection. Only bins, which contain at least are plotted. Filling colors indicate taxonomy at Class level. Each dot represents a KEGG 5 methanogenesis genes orthologue (KO) in the respective bin. Colors of the dots indicate the p-value of the log_2_FC difference between N2_MTR and H2_MTR samples. Horizontal dashed red lines mark the log_2_FC thresholds for significantly different KOs (respective p-value < 0.05)

The enrichment analysis revealed that the CH_4_ metabolism was the most affected, hence the contribution of each individual MAGs was examined next to gain a deeper insight to the molecular mechanism. An overall of 103 genes of the 8 Archaea MAGs from this pathway were down-regulated (log_2_FC lower than − 2), and 37 that were up-regulated (log_2_FC higher than 2), but of these only 61 were found to be significantly differentially expressed based on the p-value threshold of 0.05. MAGs harboring more than five KEGG map00680 pathway genes were plotted in Fig. 4.

The two MAGs identified as belonging in the genus *Methanobacterium* (bin_35 and bin_51) and *Methanoculleus* (bin_6, bin_60 and bin_66) showed a very similar response (Fig. 4), many of their map00680 genes were expressed at log_2_FC higher than 2, i.e., four-times higher expression. Two additional *Methanoculleus* MAGs (bin_60 and bin_66), a low and a medium quality MAG according to CheckM, were identified but not presented in Fig. 4. This implies that several *Methanoculleus* strains actively participate in the Power-to-Gas (P2G) reaction.

The expression level of numerous genes increased shortly after H_2_ injection in the hydrogenotrophic strains, which indicated that several metabolic pathways responded to the increased H_2_ concentrations. The log_2_FC values of the genes ENO (phosphopyruvate hydratase, EC 4.2.1.11), COF (7,8-didemethyl-8-hydroxy-5-deazariboflavin synthase, EC 4.3.1.32), and COM (sulfopyruvate decarboxylase, EC 4.1.1.79) were the largest in *M. bourgensis* i.e., 5.06, 4.2 and 1.25, respectively. The ENO enzyme takes part in the biosynthesis of the Coenzyme B, which is an essential molecule in the final step of the methanogenesis. The COF enzymes are responsible for the synthesis of the other important coenzyme, Coenzyme F_420_. The COM enzymes catalyze the 3-sulfopyruvate to 2-sulfoacetaldehyde reaction, which is an intermediate step in the synthesis of the third important coenzyme, Coenzyme-M [38]. These results clearly suggested that the cells increased the synthesis of all coenzymes, which were involved in methanogenesis to support the quick conversion of H_2_ and CO_2_ to CH_4_.

In the MAGs belonging *Methanobacterium* strains, the expression level of the enzymes MFN (tyrosine decarboxylase, EC 4.1.1.25), ADC (aspartate 1-decarboxylase, EC 4.1.1.11), FMD/FWD (formylmethanofuran dehydrogenase, EC 1.2.99.5), AKS (methanogen homocitrate synthase*,* EC 2.3.3.14 2.3.3), COM increased. These enzymes also play an important role in the hydrogenotroph methanogenesis pathway (Additional file 3: Figure S3). The MFN and ADC enzymes are normally involved in the methanofuran biosynthesis pathway, when they catalyze the l-tyrosine to tyramine reaction. The FMD/FWD redox enzyme complex contains a molybdopterin cofactor and numerous [4Fe-4S] clusters in order to catalyze the reversible reaction the formyl-methanofuran synthesis from methanofuran, which is an important methanogenesis step in CO_2_ conversion and the oxidation of coenzyme-M to CO_2_. The reaction is endergonic and is driven by coupling the soluble CoB-CoM heterodisulfide reductase via electron bifurcation. The AKS enzyme also takes part in the synthesis of Coenzyme-B.

Overall, the results signified that the hydrogenotrophic methanogenic cells activated a large number of the key enzymes in the methanogenesis pathway to consume more effectively the H_2_ from the environment. It is noteworthy that the genes of the MCR enzymes (methyl-coenzyme M reductase, EC 2.48.4.1.) showed lower expression in all hydrogenotrophic bins. The MCR enzymes (methyl-coenzyme M reductase) catalyze the final step of the methanogenesis (Additional file 3: Figure S3). One of the possible considerations explaining this observation could have been that 2 h was not enough for redirecting this section of methanogenesis pathways. If the local substrate availability did not increase significantly, the cells did not need to increase the transcriptional activity of the MCR enzymes (Additional file 4: Figure S4).

Almost all genes in *Methanosarcina honorobensis* showed decreased expression in the presence of H_2_ (Fig. 4). This strain has been described as acetotrophic, which also grew on methanol, dimethylamine, trimethylamine, dimethylsulfide and acetate but not on monomethylamine, H_2_/CO_2_, formate, 2-propanol, 2-butanol or cyclopentanol [41]. The expression levels of MCR, ACS (acetyl-CoA decarbonylase/synthase, EC 3.1.2.1) and FAE (5,6,7,8-tetrahydromethanopterin hydro-lyase, EC 4.2.1.147) significantly decreased. The ACS enzyme is responsible for the conversion of acetate to acetyl-CoA, which is a typical step in the acetotrophic methanogenesis pathway. The next enzyme, FAE generates 5,10-methylene tetrahydromethanopterin (5,10-Methylene-THMPT) from formaldehyde, an important intermediate of methanogenesis.

The substantial decrease in the transcriptional response of *M. honorobensis* to H_2_ injection corroborated that this strain is unable to utilize H_2_ and signaled an active inhibitory role of H_2_ on acetotrophic methanogenesis. This implicates a hitherto unrecognized tight regulatory role of H_2_ on diverse pathways coupled to methanogenesis (Fig. 4).

### qPCR validation of the transcriptomic data

**Fig. 5:**
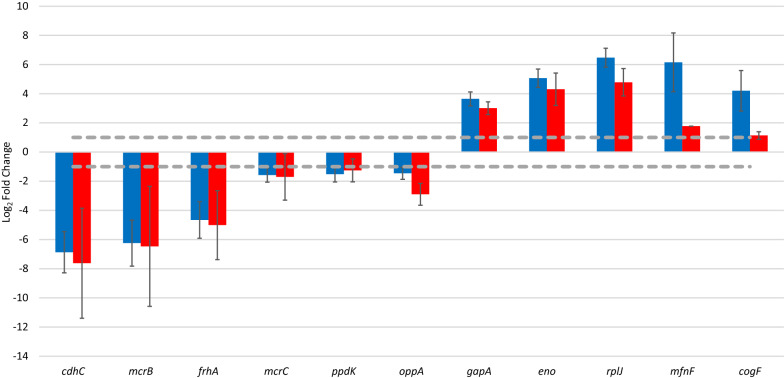
Comparison of metatranscriptomic and qPCR results of selected genes affected by early H_2_ treatment. The threshold value of significant gene expression was set to fold change 2 in gene expression (log_2_FC = 1). The selected genes are from bin_1: *ppdK* (pyruvate, phosphate dikinase); bin_6: *mfnF* ((4-{4-[2-(gamma-L-glutamylamino)ethyl]phenoxymethyl}furan-2-yl)methanamine synthase), *cofG* (7,8-didemethyl-8-hydroxy-5-deazariboflavin synthase), *rplJ* (ribosomal protein L10), *eno* (enolase); bin_27: *cdhC* (acetyl-CoA decarboxylase/synthase), *mcrB* (methyl-CoM reductase beta subunit), *frhA* (Coenzyme F_420_ hydrogenase subunit alpha); bin_35: *mcrC* (methyl-CoM reductase gamma subunit); bin_59: *gapA* (glyceraldehyde 3-phosphate dehydrogenase), *oppA* (peptide nickel transport system substrate binding protein). Blue columns: metatranscriptomic expression, red columns: qPCR results

Eleven genes were selected for testing the metatranscriptomic data by Real-Time quantitative polymerase chain reaction (RT-qPCR). The genes were selected to cover a broad range of genes displaying various gene expression levels and significant (p < 0.05) expression change according to the metatranscriptomic data. Genes participating in methanogenesis as well as others involved in cell metabolism were included. Based on the log_2_FC values (Fig. 5) most of the examined genes showed consistent results with the metatranscriptomic data, although in several cases their fold change was slightly lower than derived from the metatranscriptomic analysis. The slight deviation could have been the result of the differences between the distinct evaluation methods. The fold change (FC) calculation in the qPCR experiments is done on the traditional way (log_2_FC = log_2_(treated/control). The DESeq2 program employed in metatranscriptomics uses a series of mathematical transformations to normalize the log_2_FC values [71]. Despite the minor differences, the RT-qPCR data clearly corroborated the MTR results. Despite the minor differences, the RT-qPCR data clearly corroborated the MTR results.

Three genes may deserve special attention. The *cofG* gene had a more than 4 times smaller gene expression fold change in the qPCR experiment and it apparently diminished into the “unchanged”. category. Similarly, the FC of *mfnF* also droppped threefold although it still remained “upregulated” according to qPCR. It should be noted that DESeq2 attempts to filter the biologically relevant changes from the background noise thus in the case of *cofG* and *mfnF* the DESeq2 algorithm overestimated the FC values. The *oppA* decreased from “unchanged” to “downregulated”. This gene is a substrate-binding protein, responsible for the transport of various oligopeptides across the cell membrane [42]**.**

### Interactions between methanogenesis and other metabolic processes

**Fig. 6:**
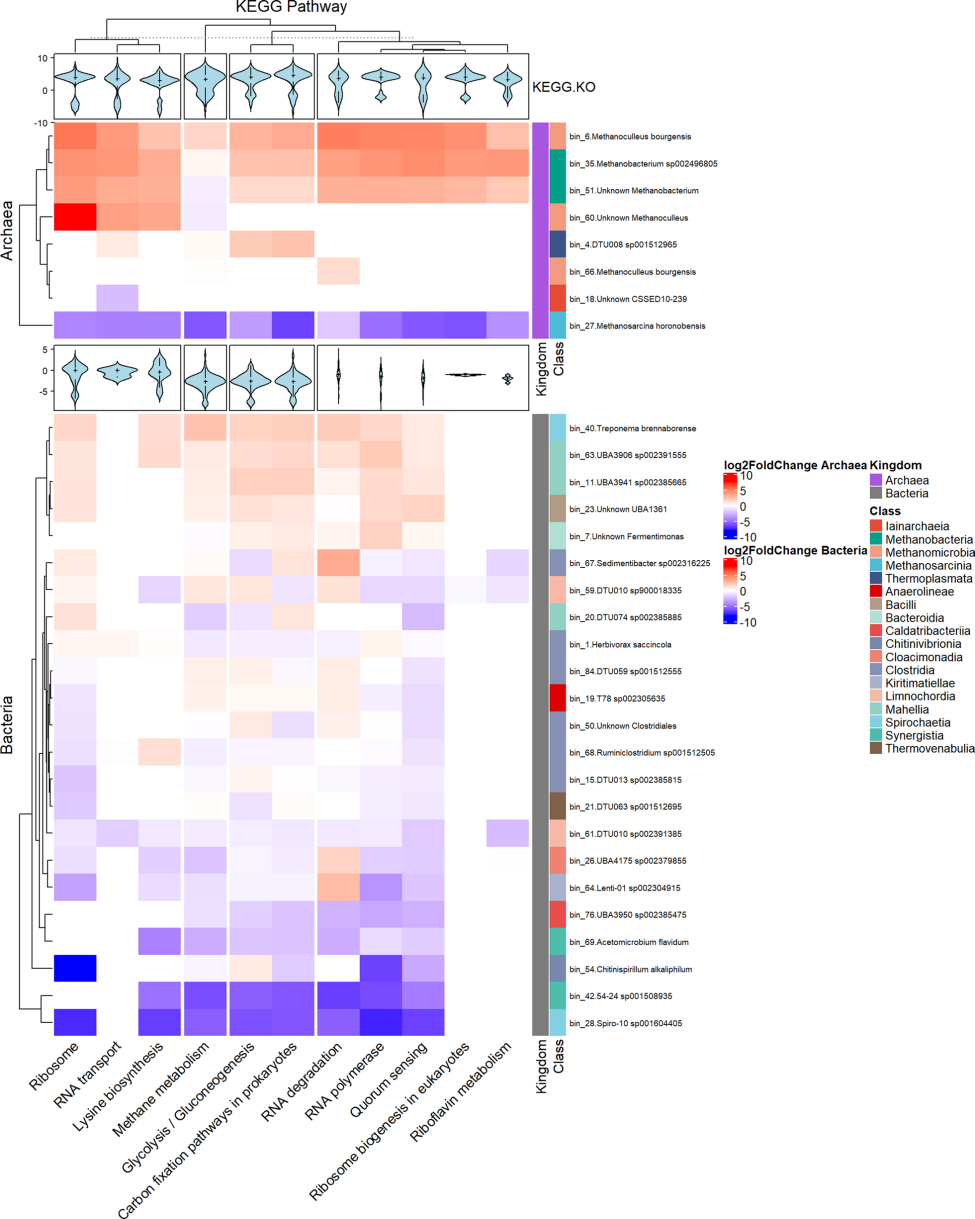
Heatmap of significantly various KEGG Pathways in bins that harbor a total of at least 10 genes in any of these pathways or modules. Top panel shows Archaea, while the bottom panel shows Bacteria bins. Filling colors are according to the log_2_FC of all the genes in that pathway/module in the given bin. Violin plots represent log_2_FC values of every gene participating in the given pathway/module

In addition to the methanogenesis pathways in the Archeal bins, we identified nine additional pathways that were expressed differently as the early response of the microbiota to H_2_ injection (Fig. 3B). Figure 6 presents the Archaea and Bacteria bins that indicate substantial up- or down regulation of several KEGG pathways. It is clear that H_2_ addition rapidly caused gene expression changes in the Archaea, i.e., bin_6, bin_27, bin_35 and bin_51, since the Ribosome, RNA polymerase and Methanogenesis pathways were altered mainly in these bins.

In the case of Archaea, one *Methanoculleus* bin (bin_6) and the two *Methanobacteria* bins (bin_35 and bin_51) responded with elevated gene expression in all pathways, while the *Methanosarcina* (bin_27) and Iainarchaeia (bin_18) responded with a substantial and general loss of transcripts, i.e., biological activity, in them.

Interestingly, the three *Methanoculleus* bins responded differently to the H_2_ injection. Apparently, the entire metabolic activity, including all KEGG orthologs, were tuned up in bin_6 (classified as *M. bourgensis*), whereas only Ribosomal activity, RNA transport and Lysine biosynthesis was strongly upregulated in bin_60 and hardly any change in metabolic activity took place in bin_66 representing presumably a separate strain of *M. bourgensis*. Their overall gene expression did increase (log_2_FC of 2.19 and 2.37, respectively), thus the observed differences might as well indicate a slower response by bin_60 and bin_66 and perhaps further H_2_ addition would have triggered a response more similar to that of the abundant *M bourgensis* (bin_6). If future experiments corroborate this situation, than the observation may indicate the time resolution limit of H_2_ triggered transcription and metabolic changes. It seems that the whole RNA machinery must be altered for responding to a significant change in the environment. Indeed, almost all genes (including the subunits of RNA polymerase for instance) from these pathways were highly expressed in the *Methanomicrobia* and *Methanobacteria* bins, and 64% of them with a log_2_FC of 2 or higher (p-value of 0.05 or lower). The early response to H_2_ injection by *Methanosarcina horonobensis* (bin_27) was quite the opposite as the expression of all investigated KEGG orthologs and metabolic pathways were hindered significantly, i.e., up to 33% (Fig. 6).

Other carbon metabolism-related pathways that showed an overall significant difference in the pathway enrichment analysis were “carbon fixation” pathways in prokaryotes and “glycolysis/gluconeogenesis”, which showed a similar pattern. For example the *folD* gene of the reductive acetyl-CoA pathway (Wood-Ljungdahl pathway) was transcribed vigorously in bin_6 (*M. bourgensis*) (log_2_FC = 3.7). The relative enrichment of *Methanogenesis, acetate to methane* was overall the highest in this bin (mean log_2_FC = 3.55), this can be linked to the elevated acetotrophic methanogenesis, as there were no other major difference between the expression change in these pathways. Interestingly though, the *Methanogenesis, *CO_2_ to methane module did not increase drastically (nor did the methylotrophic module), with the exception of a handful of genes showing log_2_FC higher than 2, including methenyltetrahydromethanopterin cyclohydrolase gene in bin_6 and bin_35 (log_2_FC = 2.56 and 3.49, respectively), and some others with smaller but still significant differences, including the F_420_-non-reducing hydrogenase iron-sulfur subunit gene of bin_6 (log_2_FC = 1.32, p-value = 0.04).

### Changes in gene expression levels in bacterial bins

Some genes involved in, or related to elements of the methanogenesis pathway could be found in bacterial bins as well, e.g. *Herbivorax saccincola*, *Ruminiclostridium *sp*001512505,* two unknown *Limnochorida* and a *Mahellia* MAG. However, when inspecting the change of the methanogenesis-related KEGG orthologs in the MAGs, it became clear that these genes showed significant difference only in a few cases, i.e., their log_2_FC values were spread between the threshold lines that indicated significance. Consequently, they were involved in the overall methanogenesis, and closely related metabolic pathways (which are included in the KEGG map00680 pathway), but they did not respond to the H_2_ provision change. This was substantially different from the behavior of the Archaea MAGs, which clearly expressed their genes differently as a respond to H_2_ injection.

In the case of Bacteria, the RNA-machinery pathways (ko03010) showed an overall decrease in gene expression, with the exception of bin_40 (*Treponema brennaborense*)*,* bin_8 (*Fermentimonas massiliensis*)*,* bin_11 (*UBA3941_sp002385665*) and bin_7 (*Unknown Fermentimonas*)*.* These MAGs had low abundance, though they showed an increase in the MTR samples. The related pathways seem to be up-regulated in bin_40 and in bin_11 (mapped in class *Mahellia*, order *Caldicoprobacterales*). Most of the small and large ribosomal subunits showed log_2_FC of 2 or higher. Another member of the family *Treponemataceae* (bin_28 *Spiro-10 sp001604405*) showed a clear downregulation in all discussed pathways.

In AD, *Treponema* behave like homoacetogenes, they consume H_2_ and CO_2_ to produce acetate, hence they may compete with hydrogenotrophic methanogens [43], although not very efficiently [17]. We identified only two methanogenesis related genes in bin_28 and bin_40 (formate-tetrahydrofolate ligase and methylenetetrahydrofolate reductase NADPH), bin_40 showed an overall activity increase (log_2_FC = 2.216), indicating either that this pathway would become more active at a later time-point, or these bacteria utilize alternative catabolic activities. In a relevant observation *Treponema* abundance increased in digesters spiked with H_2_ [44], although after 90 h the signs of H_2_ stress were noted in the digester. Essential genes of the Wood-Ljungdahl (WL) pathway were apparently not expressed in bacterial bins in a recent study [30]. In contrast, in the present work we identified several bins harboring these genes, including bin_7 *(Unknown Fermentimonas*)*,* bin_8 *(Fermentimonas massiliensis*) and bin_20 (*DTU074 sp002385885*, although all of them showed low abundance (~ 0.3–1%). Interestingly, bin_20 exhibited an overall decrease, but the expression of its WL pathway genes increased. This can be attributed to the elevation of the transcriptional activity of only two genes, the *fhs* gene (formate-tetrahydrofolate ligase) and the *folD* gene (methylene-tetrahydrofolate oxidase), which are important in WL pathway (log_2_FC = 6.31 and 3.14, respectively). This response to H_2_ is thus the opposite to that of bin_40, suggesting that as acetogenic methanogenesis increased, it might have tried to compete with the Archaea for acetate. The other two potential homoacetogens, which increased their transcriptional activity (log_2_FC = 1.40 and 2.56, respectively), apparently included the *fhs* and *folD* genes as well. It was also demonstrated earlier that homoacetogenic microbes tended to increase their activity in a H_2_-fed systems [45].

## Discussion

The interest in converting the fossil fuel based energy market to renewable energy carriers is growing worldwide. This is a very positive trend to avoid threatening global climate change and associated environmental catastrophes. The overwhelming majority of renewable energy production approaches employ photovoltaics and wind power today. Both of these technologies generate electricity in an intermittent fashion. The power distribution grids are designed to harmonize electricity production and consumption continuously, these grids can operate in a fluctuating mode only with substantial energy loss. Hence, technologies to balance the fluctuations are urgently needed. A very promising solution to this problem is offered by the flexible biogas technology [46]. Biogas plants have controllable energy output to buffer the fluctuations in renewable electricity production. Moreover, with a coupled technology called Power-to-Gas (P2G), Power-to-Methane (P2CH_4_) or Power-to-Biomethane (P2bioCH_4_), biogas reactors can efficiently convert the temporarily surplus renewable electricity to biomethane (bioCH_4_). Clean bioCH_4_ is chemically indistinguishable from the fossil natural gas, therefore it can be stored and transported efficiently and inexpensively in the natural gas grids. The biotechnological route to P2bioCH_4_ requires specific microbes capable of converting H_2_ + CO_2_ to CH_4_ in a carbon neutral or negative carbon footprint process. The key potential player microbes are methanogenic Archaea, a group of rare and obscure obligate anaerobic microbes. The precise biochemical events leading to CH_4_ formation are only understood in a broad sense today.

Understanding of the molecular regulation and control of the highly complex cell factory pathways of microbial communities carrying out AD, is a challenge for both basic and applied research. In this study we aimed at mapping the early response of the entire community, with particular attention to methanogens, a scenario frequently envisaged and expected in the P2bioCH_4_ industry [35].

In a recent study thermophilic biogas reactors were fed with H_2_, and after 18 h and 36 days MTR analyses were carried out to unveil the involvement of the individual MAGs in the global microbiome functions [30]. The results revealed a multi-trophic role to *Methanosarcina thermophila*, although the hydrogenotrophic *Methanoculleus thermophilus* prevailed as the dominant Archaea species in terms of relative gene expressions, at the expense of *M. thermophila*. Some community members emerged in the later stages of methanogenesis were below the detection limit in the starting sample, i.e., *Methanobacteriaceae* spp.

The changes in the metatranscriptome of an AD community triggered by H_2_ addition were studied before in thermophilic reactors, but the short-term response at mRNA-level to H_2_ was not Mapping the early response of the microbial community via genome-centric metatranscriptomics is therefore important for understanding and managing the turn-on and turn-off steps of the P2bioCH4 process. Genome-centric MG linked MTR investigations enables the distinction of the activity of each individual MAG and the identification of the key and most sensitive members of the community.

In the examination of the initial response of the complex AD microbial community and assessment of the first up- or down-regulated genes by the H_2_ injection a custom bioinformatics workflow was employed for the downstream analysis of the genes and pathways of each MAGs. This involved primarily the SqueezeMeta [47] pipeline, which can jointly analyze MG and MTR sequencing data. In addition a more extensive binning procedure, a subsequent pathway enrichment analysis and statistical evaluation of the log2FC of the gene expressions of the MAGs between the H_2_ and N_2_ MTR samples were carried out. In order to gain higher statistical confidence in the results, we used biological duplicates separated by a 2-month interval in CSTR AD reactors. The following important considerations were also adopted: (1) The metagenomes of the samples separated by just a 2-h time-window, i.e., before H_2_ addition and 2 h later, to make sure an unaltered microbial community. (2) qPCR tests of a handful of selected genes validated the results from the metatranscriptomics pipeline.

First we established that the composition of the microbial community did not change significantly (Fig. 2), therefore the different reproduction rates of the various taxa did not disturb the picture of early functional response. Up-to-date metagenomic and metatranscriptomic methods were employed to determine the biochemical events taking place as the result of H_2_ administration. The reproducibility of the system was tested by repeated H_2_ injections 2 months apart. Practically identical results were obtained (Fig. 2).

Four metagenome (MG) sequencing datasets were combined to assemble a fairly large number of bins (84 bins: seven Archaea, 61 Bacteria and 16 unclassified bins). The non-H_2_-adapted, “raw” biogas forming microbial community was essentially the same in structure and composition as the ones sampled previously from the same industrial biogas plant fed with manure and maize silage [48, 49]. This community switched to H_2_ consumption and biomethane production almost immediately following H_2_ injection, although feeding of the entire community with alpha-cellulose substrate continued as before. We interpret that this behavior indicated the presence of sufficient hydrogenotrophic methanogenesis activity in the “raw” biogas community, i.e., in the large scale biogas plant effluent, to perform the P2bioCH_4_ conversion at full speed. In other words, the diverse, “raw” anaerobic communities can be used in switching on P2bioCH_4_ without a lengthy adaptation and enrichment period. This allows a quick and efficient turn-on and turn-off response by the mixed methanogenic community. The microbial community composition rearranges upon long-term exposure to H_2_ (and CO_2_), particularly when no other organic substrate is available for the community [49]. The vigorous P2bioCH_4_ activity returned to normal biogas production as soon as the dissolved H_2_ diminished, but the community was ready to adjust its biochemistry to instant H_2_ conversion and P2bioCH_4_ repeatedly.

The metatranscriptomic responses to the H_2_ treatments separated 2 months apart were very similar to each other indicating that the metabolic pathways were flexibly restored after switching on and off the P2bioCH_4_ operational mode. A thorough analysis of the differences between the H_2_-treated metatranscriptomes and corresponding controls identified the early events in the microbial communities brought about by H_2_.

H_2_ (and dissolved CO_2_) is readily converted to CH_4_ by both direct (hydrogenotrophic) and indirect (homoacetogenesis and subsequent acetotrophic) methanogenesis. Our results suggest that the second route is unlikely the predominant one in the early response of the microbial community to H_2_ addition at least under mesophilic conditions, since the acetotrophic pathways reacted sluggishly, while the gene transcription of the hydrogenotrophic route increased dramatically after a very short period of extensive H_2_ feeding (Figs. 4, 5). This predicts that under the P2bioCH_4_ operation conditions the physiological readiness of the hydrogenotrophic methanogen members of the community will determine the reactor response rate upon switch-on of the H_2_ addition.

Interestingly, this study revealed an extensive reaction to the transient H_2_ stress within the Bacteria community as well although Bacteria cannot directly generate CH_4_ from H_2_ as many Archaea can. Some of these Bacteria possess the complete or partial enzyme sets for the Wood-Ljungdahl pathway. These and the homoacetogens are probably the best candidates for syntrophic community interactions between members of the distinct kingdoms of Archaea and Bacteria. The details of these interactions in the complex anaerobic environment and consequences to stabilize robust and vigorous P2bioCH_4_ microbial communities during long term P2G operation should be the subjects of future studies. Nevertheless, the transcriptional activity of the primary potential syntrophic bacterial partners (bin_1 (*Herbivorax saccincola*), bin_68 (*Ruminococcus *sp.), and unidentified bins_59, _61, _63, see Fig. 4) did not change substantially upon H_2_ exposure. This may mean that either there is enough syntrophic capacity already in the non-adapted, “raw” community to support increased hydrogenotrophic methanogen activity or the syntrophic partners respond slowly to the sudden H_2_ burst appearing in the microbial environment.

The development of a stable P2CH_4_ community strongly depends on environmental conditions and on the starter microbial community composition. Various reactor designs, operational parameters and inocula are being tested making rigorous comparison of the results difficult.

In a brief review to summarize in situ biogas upgrading Zhang et al. [50], pointed out the predominant roles of the genus *Methanoculleus* under mesophilic conditions and the thermophilic genus *Methanothermobacter* at elevated temperatures. The species *M. bourgensis* (bin_6) was identified to play an important role in various biogas reactor systems. *Methanoculleus* species grow on CO_2_ and H_2_ and hence perform the hydrogenotrophic pathway for CH_4_ synthesis [51]. In line with these conclusions, the mesophilic AD methanogenic community of palm oil mill effluent with eventual addition of formate was predominated by members of the genus *Methanoculleus* [52]. Various inocula were compared for biomethane production at mesophilic conditions in batch fermentations. It was concluded that the abundance and activity of the genera *Methanosarcina* and *Methanoculleus* played key roles in methanogenesis of added H_2_ [52], while the authors also noted the regulatory role of the available CO_2_/bicarbonate in the production of CH_4_ and/or volatile fatty acids.

In a recent work [53] the microbial community changes were followed under various operational conditions starting from two distinct inocula, i.e., wastewater (WW) sludge and plug-flow reactor operated with agricultural waste (PF). The study pointed out the importance of the history of the inoculum communities. In the WW inoculated batch reactors the methanogenic genus *Methanobacterium* and *Methanothrix* predominated and upon H_2_ feeding the genus *Methanobacterium* took over. In the plug-flow reactor, supplied with animal manure and ensilaged plant biomass, the initial abundance of genus *Methanothrix* diminished and the methanogenic gap was filled in by members of the genera *Methanobacterium* and *Methanoculleus*. This study corroborated the previous observations [19, 30] concerning the regulatory role of H_2_ concentration and CO_2_ depletion in the selection of hydrogenotrophic methanogens predominating the P2bioCH_4_ community.

In a thorough in situ syngas bioconversion study running two UASB reactors in sequence at mesophilic temperature [54], observed the predominance of the genus *Methanotrix* (formerly *Methanosaeta*). The reactors were continuously fed with varying glucose loads. *Methanotrix* species apparently cannot carry out hydrogenotrophic methanogenesis, therefore their predominance under these conditions can be rationalized by the combined effects of glucose and CO-rich syngas addition via carboxydotrophic methanogenesis [55]. In addition, the recently recognized capability of *Methanotrix* species to carry out direct electron transfer (DIET) to drive CO_2_ reduction could facilitate the *Methanotrix* predominance [56, 57].

Taking into account the recent results and considerations, the development of a stable P2bioCH4 mixed AD community depends on a number of important parameters, such as the origin of inoculum, H_2_ supply and its fluctuation, composition of added growth supporting substrates, the dissolved CO_2_/HCO_3_^−^ concentration, temperature and reactor configuration. In the future the extension of these studies should be carried out, i.e., mapping the molecular events after longer exposure of the microbial cell factory and linking the metagenomic approach to more detailed transcriptomic and proteomic studies.

## Conclusion

In this study the early response of the mixed biogas microbial community to the presence of saturating amount of H_2_ was examined. Metagenomic and metatranscriptomic analyses have been carried out to determine the changes of the expression levels of the various genes related to methanogenesis. The results indicated that the microbial community responded instantaneously to the presence of H_2_. The activity of acetotrophs reduced significantly. In addition, the metabolic activity of numerous bacterial strains changed substantially as a response to H_2_. Clearly, the excess H_2_ does not only affect the methanogenesis pathways in Archaea, rather the microbial community respond with a multifarious gene expression profile change, which seems to be rather selective. This indicates a more global regulatory role of H_2_ in the life of anaerobic communities than assumed earlier. The syntrophic interactions contribute to the stability and metabolic activity of the hydrogenotrophic methanogens. This, together with the non-sterile operation conditions and continuous supply of inexpensive catalyst, underlines the benefits of using mixed communities in the P2bioCH_4_ process instead of pure hydrogenotrophic cultures [35, 58, 59].

## Materials and methods

### Anaerobic fermentation

Anaerobic digestions (AD) were carried out in continuously stirred tank reactors [60]. The fermentation volume was 5000 mL, leaving a headspace of 2000 mL. The apparatus can be fed continuously or intermittently via a piston type delivery system, the fermentation effluent is removed through an air-tight overflow. The reactors are equipped with a spiral strip mixing device driven by an electronic engine. An electronically heated jacket surrounds the cylindrical stainless steel body, electrodes for the measurement of pH and redox potential are inserted through the reactor wall, in sealed sockets. The device can be drained at the bottom. The evolved gas leaves the reactor through the top plate, where ports for gas sampling and the delivery of liquids by means of syringes through silicone rubber septa are also installed. Gas volumes are measured with thermal mass flow devices (DMFC SLA5860S, Brooks). A fresh sample from an industrial scale mesophilic biogas plant, fed with pig slurry and maize silage mix (Zöldforrás Biogas Plant, Szeged, Hungary) was used as an inoculum, i.e., the microbial community adopted to heterogeneous substrate degradation. The reactors were flushed with N_2_ to ensure anaerobic conditions and were closed air tight. During the experiment the digesters were fed twice a day with synthetic medium in which only alpha-cellulose was added as a carbon source at a loading rate of 1 g oDM L-1 day-1. The reactors were operated under mesophilic conditions, at 37 °C.

### Determination of fermentation parameters

Organic dry matter (ODM): The dry matter content was determined by drying the biomass at 105 °C overnight and weighing the residue. Further, heating of this residue at 550 °C provided the organic total solids content.

NH_4_^+^–N: For the determination of NH_4_^+^–N content, the Spectroquant Ammonium test (1.00683.0001 test, Merck, Kenilworth, N.J, USA) was used in a Nova 60 spectrophotometer according to the manufacturer’s instructions.

VOA/TIC (Volatile organic acids/Total inorganic carbon): 5 g of each AD samples were taken for analysis and diluted appropriately with distilled water. The subsequent measurement was carried out with a Pronova FOS/TAC 2000 Version 812-09.2008 automatic titrator (Pronova, Germany).

### Sampling

The first set of samples were taken when the reactor operation was stabilized under N_2_ in the headspace, the daily biogas production, CH_4_ content and total organic acid/buffer capacity ratio were constant. 2 mL of reactor content was withdrawn and total RNA for transcriptome analysis (sample names: N2-MTR) and DNA for metagenome analysis (sample names: N2_MG) were isolated immediately after sampling. Than the digesters were flushed with pure H_2_ gas for 10 min on day 15 and 71. H_2_ was injected directly from a pure H_2_ gas cylinder through custom made nozzles (10 pieces) having 0.2 mm holes. The applied gas pressure was 2 bar, the gas purity was 99.999%. 2 h after flushing the reactors with H_2_ samples were also taken for RNA (sample names: H2_MTR) and DNA (sample names: H2_MG) isolation. The headspace was then replaced with N_2_ and the reactors were run under the same conditions as before. After 2 months the whole H_2_ treatment procedure was repeated in order to test the reproducibility of the set-up. At the sampling time points two biological parallels were withdrawn.

### RNA isolation and cDNA synthesis

For RNA isolation 2 mL of reactor liquid samples were used. The samples were centrifuged at 12,000 rpm for 10 min. RNA extractions were carried out with the Zymo Research Soil/Fecal RNA kit (R2040, Zymo Research, Irvine, CA, United States). After lysis (bead beating), the Zymo Research kit protocol was followed. The DNA contamination was removed by Thermo Scientific Rapidout™ DNA removal kit (K2981, Thermo Fisher Scientific, Waltham, MA, United States). Ribosomal RNA was depleted using the Ribo-Zero™ rRNA Removal Kit for Bacteria (Illumina, Madison, USA) according to the manufacturer’s instructions. The rRNA depleted samples were purified via the RNA Clean and Concentrator Columns from Zymo Research (Irvine, USA). During this step, an additional in-column DNase I treatment was included to ensure complete removal of DNA. Subsequently, synthesis of double-stranded cDNA was conducted using the Maxima H Minus Double-Stranded cDNA Synthesis Kit from ThermoScientific (Waltham, USA). In the first-strand cDNA synthesis reaction, 2 μL of random hexamer primer were used. Final purification of the blunt-end double-stranded cDNA was carried out using SureClean Plus solution from Bioline (Luckenwalde, Germany). The cDNA was sequenced in the same way as the total DNA. The quality of the RNA preparation was checked by agarose gel electrophoresis (data not shown).

### DNA isolation

DNA extractions were carried out from 2 mL reactor liquid using a slightly modified version of the Zymo Research Fecal DNA kit (D6010, Zymo Research, Irvine, USA). The lysis mixture contained 100 µL of 10% CTAB (cetyltrimethylammonium bromide) to improve the efficiency [61]. After lysis (bead beating was performed by Vortex Genie 2, bead size: 0.1 mm; beating time: 15 min, beating speed: max) and subsequently the Zymo Research kit protocol was followed.

The quantity of DNA was determined in a NanoDrop ND-1000 spectrophotometer (NanoDrop Technologies, Wilmington, DE, United States) and a Qubit 2.0 Fluorometer (Life Technologies, Carlsbad, CA, United States). DNA purity was tested by agarose gel electrophoresis and on an Agilent 2200 TapeStation instrument (Agilent Technologies, Santa Clara, CA, United States).

### Sequencing

Paired-end libraries were prepared for the metagenome and metatranscriptome samples using the NEBNext^®^ Ultra™ II DNA Library Prep Kit for Illumina (Cat.Num.: E7645L). Paired-end fragment reads were generated on an Illumina NextSeq sequencer using TG NextSeq^®^ 500/550 High Output Kit v2 (300 cycles). Raw read sequences (*.fastq* files) are available on NCBI-SRA under the following BioProject ID: PRJNA 698464.

### Reverse transcription coupled quantitative PCR

Elevenmpared with the Bio-Gas Microbiome dat genes were selected for reverse transcription coupled quantitative PCR (RT-qPCR) based on the metatranscriptomic data. From every sample, 500 ng of RNA was transcribed into cDNA with the High Capacity cDNA Reverse Transcription Kit (Thermo Fisher Scientific, Waltham, MA, USA) according to the instructions of the manufacturer. The PCR primer oligonucleotides were synthesized by Eurofins Genomics (Eurofins Genomics, Ebersberg, Germany). The primers are listed in Additional file 6: Table S2. The reactions were prepared in a final volume of 25 µL with Kapa SYBR Fast Universal qPCR kit (Roche, Basel, Switzerland). The qPCR experiments were carried out on a BioRad CFX96 Touch Real-Time PCR Detection System (BioRad, Hercules, CA, USA) with the following parameters: initial denaturation was done at 95 °C for 3 min then 40 cycles of 95 °C for 10 s and 60 °C for 30 s. For quantification of the gene copies, standards were prepared with every primer pair from the genomic DNA. The standards were amplified with DreamTaq DNA Polymerase in a BioRad T100 Thermal Cycler with the following parameters: initial denaturation was at 95 °C for 3 min, then 35 cycles of 95 °C for 30 s, 60 °C for 30 s, 72 °C for 20 s. After amplification, the PCR products were purified with Viogene PCR Advanced PCR Clean Up Miniprep System (Viogene Biotek Corp., New Taipei City, Taiwan) following the manufacturer’s instructions. The PCR product concentration was determined on a Qubit4 fluorimeter (Thermo Fisher Scientific) with a Broad Range Assay Kit. The molarity of the PCR products was calculated based on the size and concentration of the particular gene fragment. Dilution series were created from the PCR products with a factor of 10 from 1 × 10^9^ to 1 × 10^1^ copies µL^−1^. The dilution series were measured on the same plate with their corresponding cDNA samples in the RT-qPCR experiments. The RT-qPCR runs were evaluated with CFX Maestro version: 4.1.2433.1219 (BioRad). log_2_FC of the gene expression was calculated as for the transcriptomics data.

### Bioinformatics

Quality filtering and trimming of the raw reads were carried out with FastQC. Assembly with MegaHIT, ORF prediction with prodigal and predicted gene functional annotation was carried out within the SqueezeMeta workflow [47]. For the KEGG KO annotation EggNOG database (v. 5) was used [62]. Binning of the contigs was carried out with four different binning algorithms: Metabat2 [25], Maxbin2 [63], Concoct [24] and Binsanity [64]. The result of each binning procedure was further improved with DAS tool [65]. Bin qualities were estimated with CheckM [36] and bin taxonomy was determined using the GTDB database. A phylogenomic tree from the protein genomes of the MAGs were built with the phylophlan3 program [66]: *phylophlan –diversity high –fast -f phylophlan_configs/supermatrix_aa.cfg -t a –min_num_markers 75*. The assembly, annotation, binning and phylogenomic results were imported into and subsequently visualized with the Anvi’o [67] platform. The results were compared with the Bio-Gas Microbiome database (Additional file 5: Table S1).

The filtered reads from each sample were mapped back onto each bins with bowtie2 [68] and FeatureCounts [69] was used to calculate the gene count table by using the ORF predictions of the bins. Since we were primarily interested in pathway analysis, genes that could be annotated with a KEGG Orthology (KO) were kept [70]. For the assessment of log_2_ fold changes (log_2_FC) between the samples the DESeq2 package was used [71], which was proven to be an appropriate method to infer differences between metagenomic and metatranscriptomic gene counts [72]. The following parameters were set: test = "Wald", fitType = "parametric", filterFun = ihw. For the assessment of significance, the Benjamini-Hochberg-adjusted p-values were used (termed ‘*padj’*), with a threshold of 0.05.

Differentially expressed KOs and pathways were assessed at two levels: First, counts of genes with the same KO annotation were grouped together and summed in each sample. Differentially expressed KOs between the two MTR samples were then determined with DESeq2 as described above. The resulting DE KO list was the input for Clusterprofiler R package [73] to detect differentially expressed pathways. Then counts of genes with the same KO annotation were grouped together in each sample and in each bin, since our main focus was to assess which pathways changed in the individual genome bins. Genes that did not belong to any bin were grouped together as *unbinned*. Differentially expressed KOs of every bin between the two MTR samples were then determined with DESeq2, based on log_2_FC and p-values. This bin-KEGGKO-sample table was also rlog-transformed (regularized logarithm transformation) with the *rlog* function of the DESeq2 package and results were subjected to a Principial Component Analysis (PCA) using the FactoMineR package.

## Supplementary Information

**Additional file 1: Figure S1.** Daily biogas productions (green spots) and their average (red line) during the experimental period. H_2_ injection took place at time points marked with dotted blue arrows. Increment CH_4_ production is highlighted with yellow curves fitted to the data points. The areas under these curves were used for CH_4_ conversion estimation.

**Additional file 2: Figure S2.** Relative abundances of Archaea and Bacteria bins.

**Additional file 3: Figure S3.** Methanogenesis enzymes affected by H_2_ addition.

**Additional file 4: Figure S4.** KEGG heatmap.

**Additional file 5: Table S1.** Comparison of bins identified using the GTDB database with Bio-Gas Microbiome database. The two bins containing high quality metagenomes, which did not find their corresponding MAGs are highlighted. Percent AAI stands for percent amino acid identity. # Indicates the bins, which were not identified by the one or both of the databases.

**Additional file 6: Table S2.** Genes and qPCR primers used in this study.

## Data Availability

All the R scripts that were used to analyze the data are available upon request. Raw read sequences (*.fastq* files) are available on NCBI-SRA under the following BioProject ID: PRJNA698464 (https://www.ncbi.nlm.nih.gov/bioproject/PRJNA698464).
