# Supplementary figures and images for: Early response of methanogenic archaea to H2 as evaluated by metagenomics and metatranscriptomics
Source: Microb Cell Fact. 2021 Jul 3;20:127. doi: 10.1186/s12934-021-01618-y (PMC8254922; doi:10.1186/s12934-021-01618-y)

## Slide 1
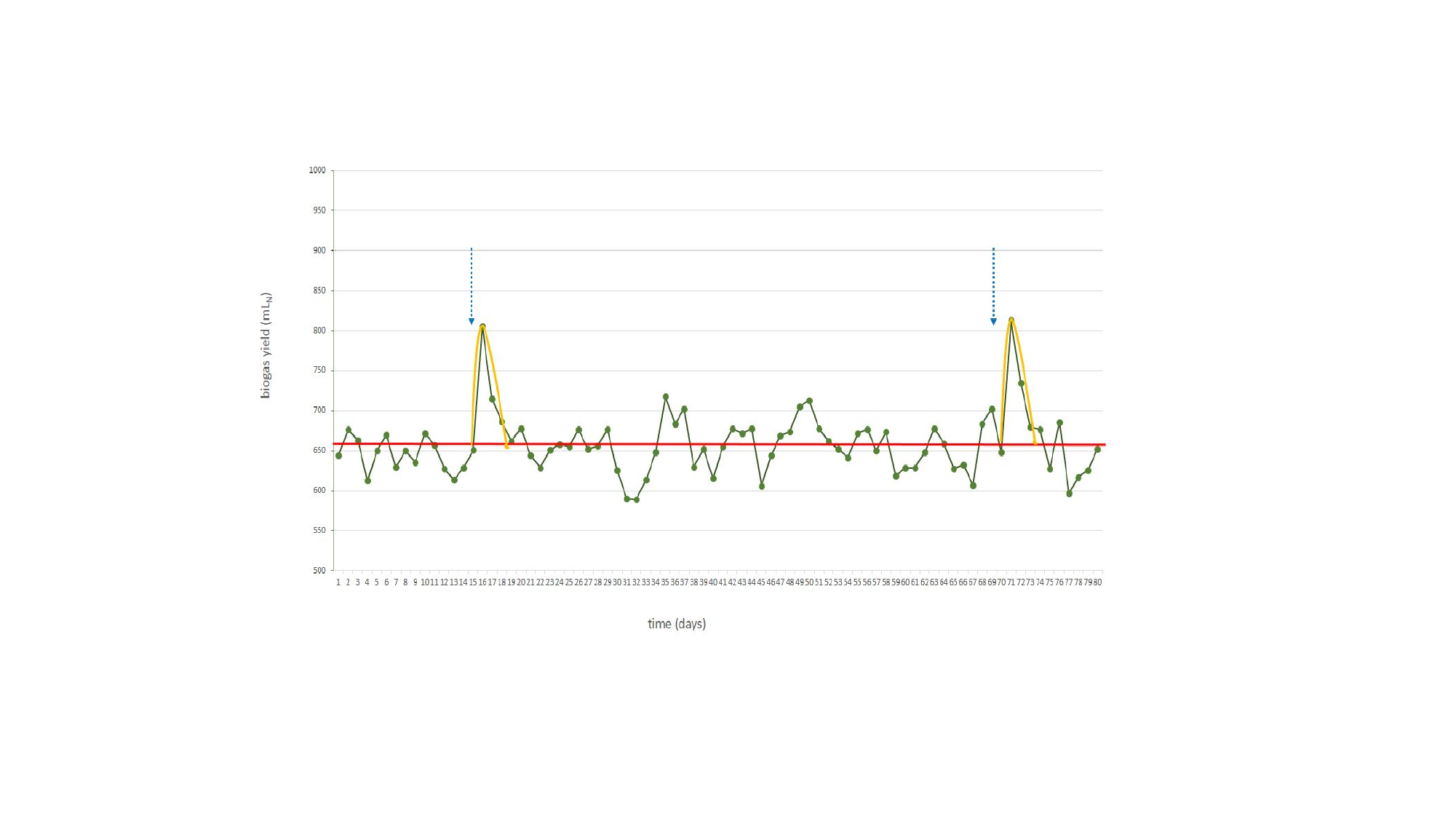

Supplement: Supplementary file 1 — Additional file 1: Figure S1. Daily biogas productions (green spots) and their average (red line) during the experimental period. H2 injection took place at time points marked with dotted blue arrows. Increment CH4 production is highlighted with yellow curves fitted to the data points. The areas under these curves were used for CH4 conversion estimation. [file 12934_2021_1618_MOESM1_ESM.pptx]

## Slide 1
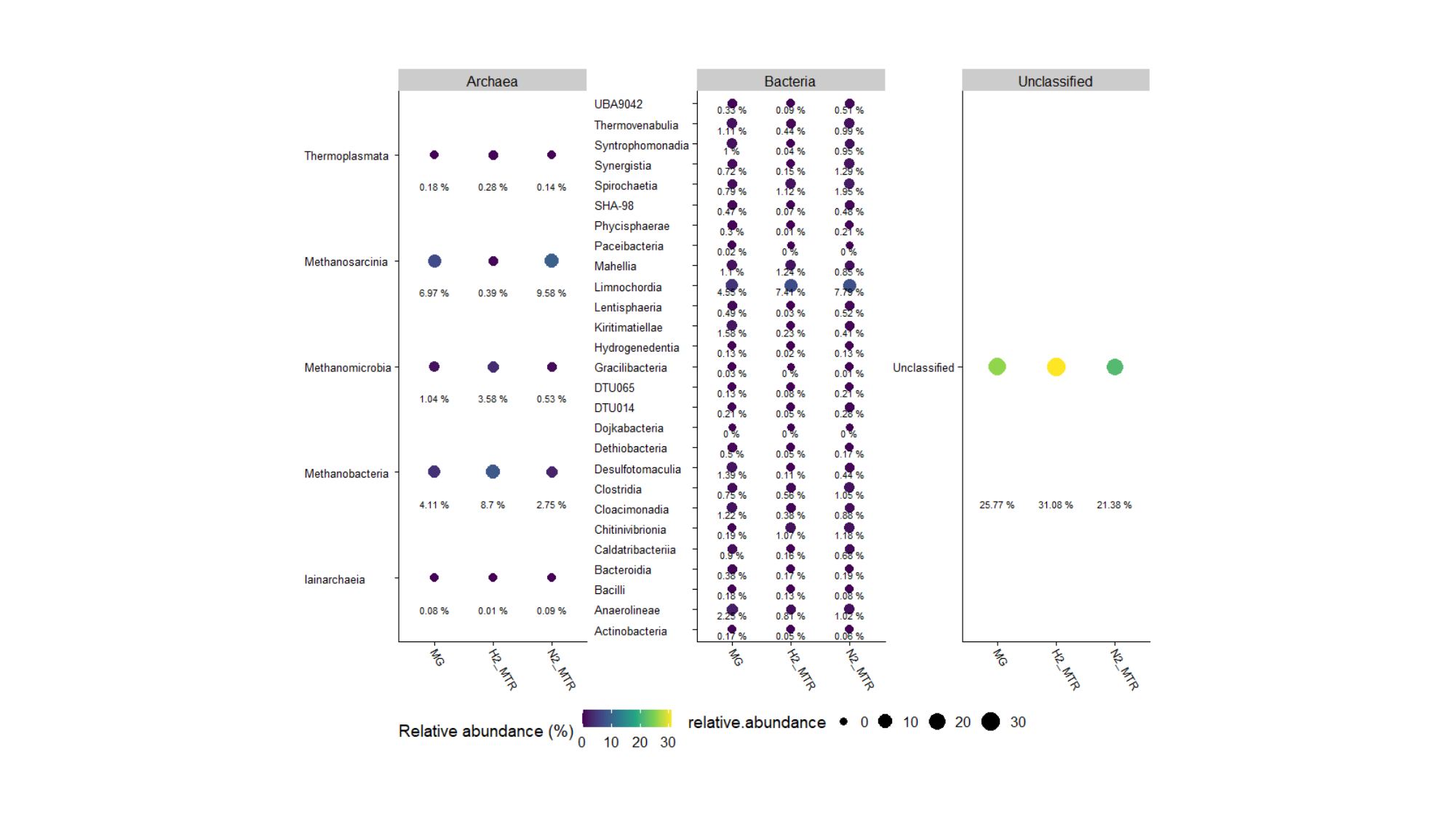

Supplement: Supplementary file 2 — Additional file 2: Figure S2. Relative abundances of Archaea and Bacteria bins. [file 12934_2021_1618_MOESM2_ESM.pptx]

## Slide 1
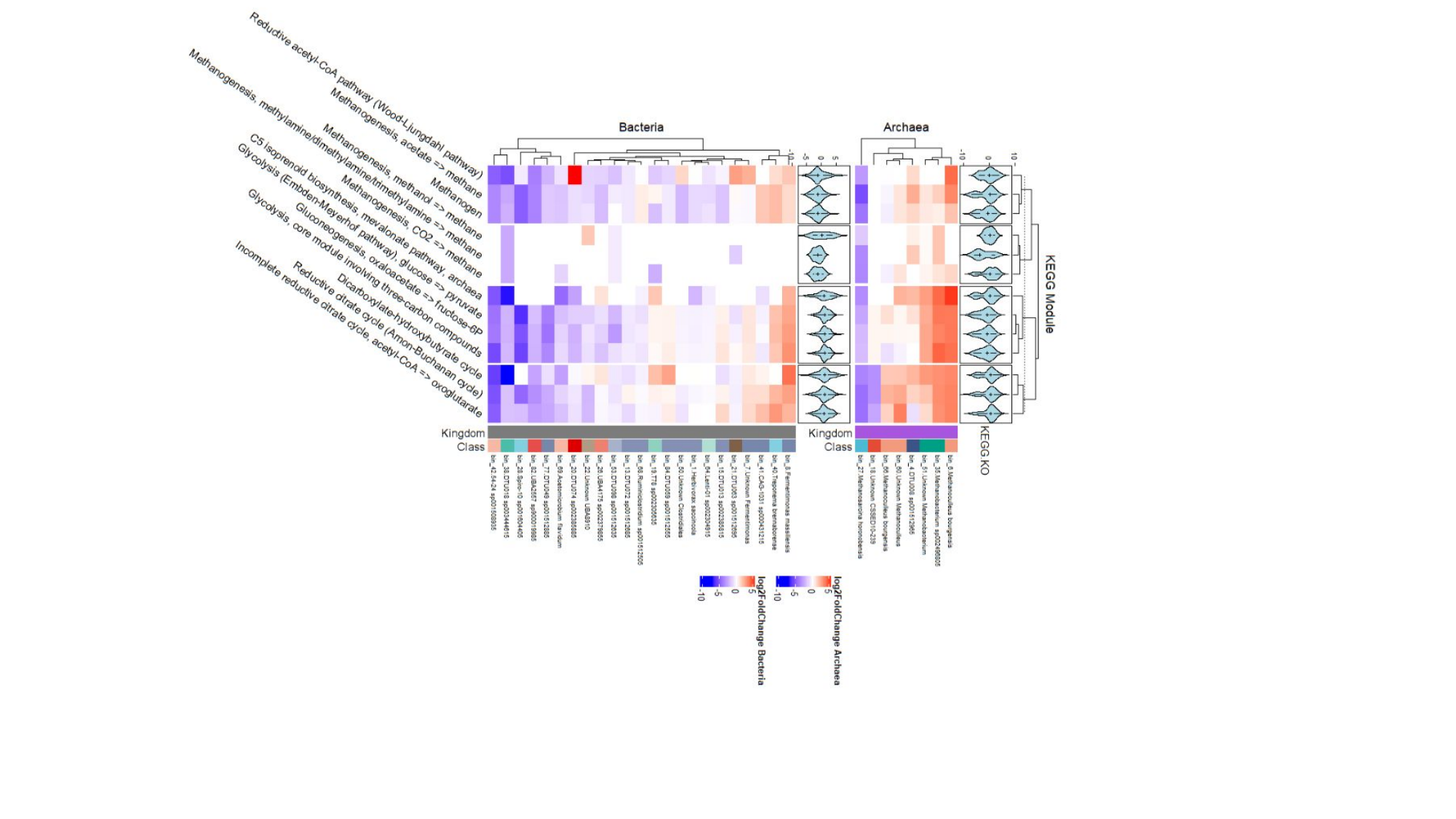

Supplement: Supplementary file 4 — Additional file 4: Figure S4. KEGG heatmap. [file 12934_2021_1618_MOESM4_ESM.pptx]
