# Supplementary material for: Early response of methanogenic archaea to H2 as evaluated by metagenomics and metatranscriptomics
Source: Microb Cell Fact. 2021 Jul 3;20:127. doi: 10.1186/s12934-021-01618-y (PMC8254922; doi:10.1186/s12934-021-01618-y)
Supplement: Supplementary file 3 — Additional file 3: Figure S3. Methanogenesis enzymes affected by H2 addition. [file 12934_2021_1618_MOESM3_ESM.pptx]

## Slide 1
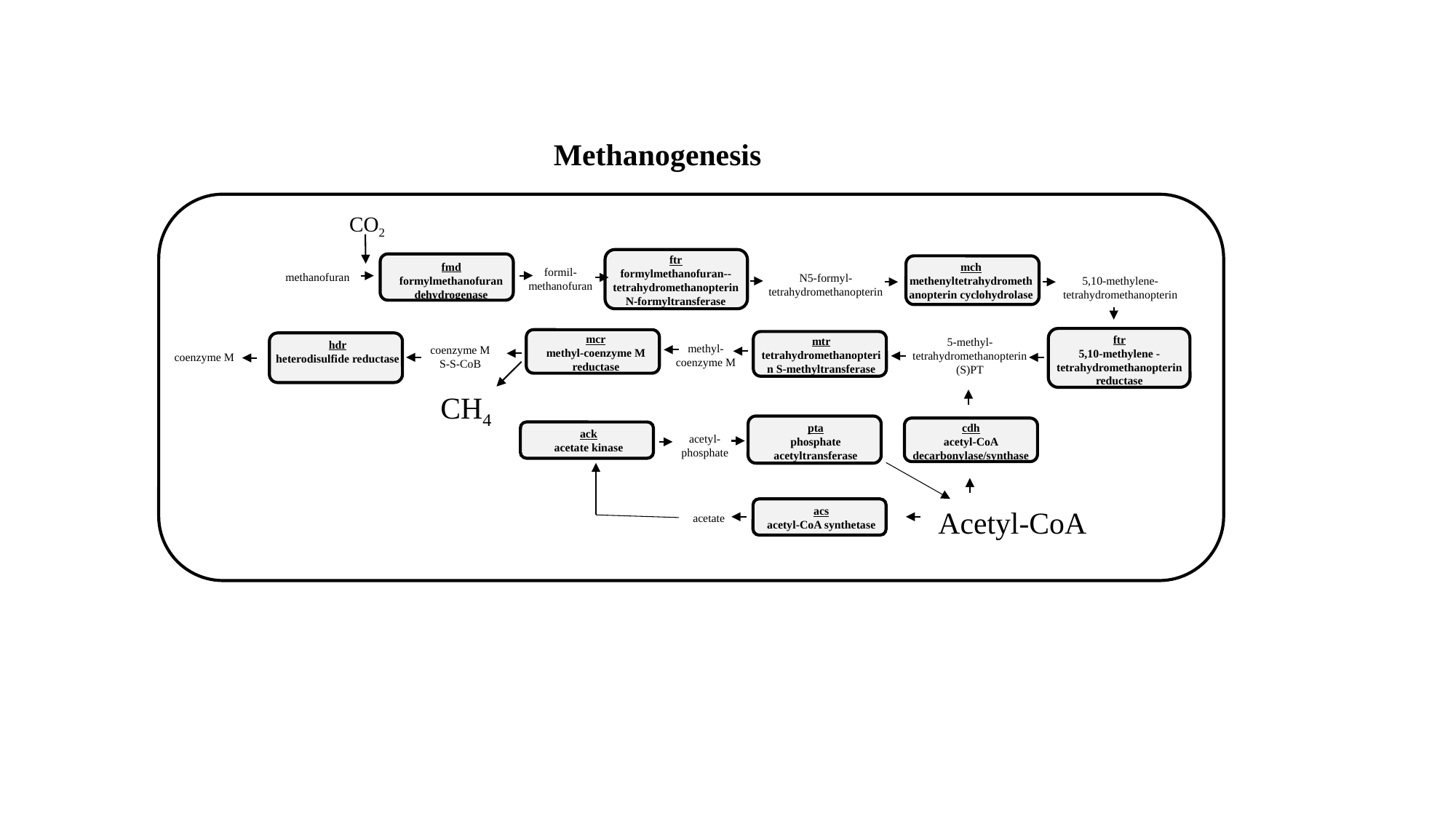

Methanogenesis
CO2
ftr
formylmethanofuran--tetrahydromethanopterin N-formyltransferase
fmd
formylmethanofuran dehydrogenase
mch
methenyltetrahydromethanopterin cyclohydrolase
formil-methanofuran
methanofuran
N5-formyl-tetrahydromethanopterin
5,10-methylene-tetrahydromethanopterin
mcr
methyl-coenzyme M reductase
ftr
5,10-methylene -tetrahydromethanopterin reductase
mtr
tetrahydromethanopterin S-methyltransferase
5-methyl-tetrahydromethanopterin(S)PT
hdr
heterodisulfide reductase
methyl-coenzyme M
coenzyme M S-S-CoB
coenzyme M
CH4
cdh
acetyl-CoA decarbonylase/synthase
pta
phosphate acetyltransferase
ack
acetate kinase
acetyl-phosphate
acs
acetyl-CoA synthetase
Acetyl-CoA
acetate
