# Supplementary material for: Early response of methanogenic archaea to H2 as evaluated by metagenomics and metatranscriptomics
Source: Microb Cell Fact. 2021 Jul 3;20:127. doi: 10.1186/s12934-021-01618-y (PMC8254922; doi:10.1186/s12934-021-01618-y)
Supplement: Supplementary file 6 — Additional file 6: Table S2. Genes and qPCR primers used in this study. [file 12934_2021_1618_MOESM6_ESM.pptx]

## Slide 1
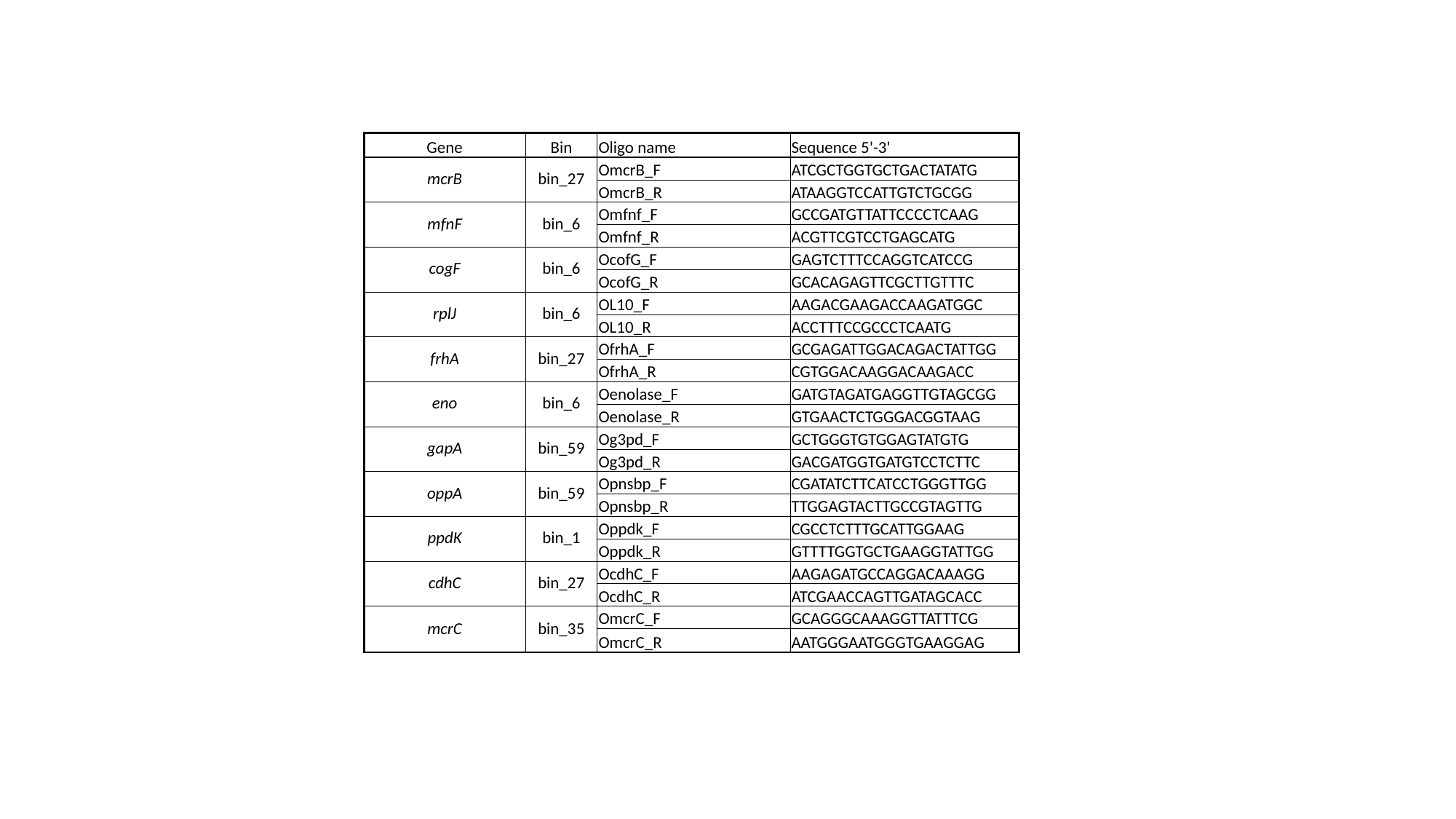

| Gene | Bin | Oligo name | Sequence 5'-3' |
| --- | --- | --- | --- |
| mcrB | bin\_27 | OmcrB\_F | ATCGCTGGTGCTGACTATATG |
| | | OmcrB\_R | ATAAGGTCCATTGTCTGCGG |
| mfnF | bin\_6 | Omfnf\_F | GCCGATGTTATTCCCCTCAAG |
| | | Omfnf\_R | ACGTTCGTCCTGAGCATG |
| cogF | bin\_6 | OcofG\_F | GAGTCTTTCCAGGTCATCCG |
| | | OcofG\_R | GCACAGAGTTCGCTTGTTTC |
| rplJ | bin\_6 | OL10\_F | AAGACGAAGACCAAGATGGC |
| | | OL10\_R | ACCTTTCCGCCCTCAATG |
| frhA | bin\_27 | OfrhA\_F | GCGAGATTGGACAGACTATTGG |
| | | OfrhA\_R | CGTGGACAAGGACAAGACC |
| eno | bin\_6 | Oenolase\_F | GATGTAGATGAGGTTGTAGCGG |
| | | Oenolase\_R | GTGAACTCTGGGACGGTAAG |
| gapA | bin\_59 | Og3pd\_F | GCTGGGTGTGGAGTATGTG |
| | | Og3pd\_R | GACGATGGTGATGTCCTCTTC |
| oppA | bin\_59 | Opnsbp\_F | CGATATCTTCATCCTGGGTTGG |
| | | Opnsbp\_R | TTGGAGTACTTGCCGTAGTTG |
| ppdK | bin\_1 | Oppdk\_F | CGCCTCTTTGCATTGGAAG |
| | | Oppdk\_R | GTTTTGGTGCTGAAGGTATTGG |
| cdhC | bin\_27 | OcdhC\_F | AAGAGATGCCAGGACAAAGG |
| | | OcdhC\_R | ATCGAACCAGTTGATAGCACC |
| mcrC | bin\_35 | OmcrC\_F | GCAGGGCAAAGGTTATTTCG |
| | | OmcrC\_R | AATGGGAATGGGTGAAGGAG |
